# Peripheral nerve injury repair by electrical stimulation combined with graphene-based scaffolds

**DOI:** 10.3389/fbioe.2024.1345163

**Published:** 2024-02-28

**Authors:** Yuanyuan Zhao, Yang Liu, Shiqi Kang, Daokuan Sun, Yufeng Liu, Xin Wang, Laijin Lu

**Affiliations:** ^1^ Department of Hand and Foot Surgery, Orthopedics Center, The First Hospital of Jilin University, Changchun, China; ^2^ Key Laboratory of Automobile Materials of MOE, College of Materials Science and Engineering, Jilin University, Changchun, China

**Keywords:** peripheral nerve injury, electrical stimulation, graphene-based scaffolds, neural tissue engineering, nerve regeneration

## Abstract

Peripheral nerve injury (PNI) is a common clinical problem, which due to poor recovery often leads to limb dysfunction and sensory abnormalities in patients. Tissue-engineered nerve guidance conduits (NGCs) that are designed and fabricated from different materials are the potential alternative to nerve autografts. However, translation of these NGCs from lab to commercial scale has not been well achieved. Complete functional recovery with the aid of NGCs in PNI becomes a topic of general interest in tissue engineering and regeneration medicine. Electrical stimulation (ES) has been widely used for many years as an effective physical method to promote nerve repair in both pre-clinical and clinical settings. Similarly, ES of conductive and electroactive materials with a broad range of electrical properties has been shown to facilitate the guidance of axons and enhance the regeneration. Graphene and its derivatives possess unique physicochemical and biological properties, which make them a promising outlook for the development of synthetic scaffolds or NGCs for PNI repair, especially in combination with ES. Considering the discussion regarding ES for the treatment of PNI must continue into further detail, herein, we focus on the role of ES in PNI repair and the molecular mechanism behind the ES therapy for PNI, providing a summary of recent advances in context of graphene-based scaffolds (GBSs) in combination with ES. Future perspectives and some challenges faced in developing GBSs are also highlighted with the aim of promoting their clinical applications.

## 1 Introduction

Peripheral nerve injury (PNI) is a common and widespread clinical disease. Acute trauma, autoimmune diseases, local lesions, and infections are all triggers for injuries in the peripheral nervous system (Asthana et al., 2021). Peripheral nerves form an extensive neural network throughout the entire body, connecting the nerve center with target organs and enabling communication between them (Wieringa et al., 2018). Therefore, when PNI occurs, the lack of information transmission poses a severe threat to the mobility and sensory function in distal target organs. Even though peripheral nerves boast the intrinsic capacity to regenerate over small gaps, the slow growth rate of about 1 mm/day results in a limited regeneration of nerve function for nerve injuries more than 3 mm in length (Sunderland, 1947; Mietto et al., 2015; Brugger et al., 2017). After injury, the recovery of the innervation for the distal target organ takes a significant amount of time, and in the case of severe injuries, the distal end of the injured nerve and target organs will atrophy over time, resulting in long-term sensory and motor dysfunction (Fu and Gordon, 1995; Zhang S. et al., 2022).

The slow regeneration speed of peripheral nerves and the poor recovery of nerve function after regeneration have posed challenges in clinical practice for numerous years. Among various treatment methods, autologous nerve transplantation has been considered the gold standard for treating long-gap injuries, albeit the successful rate of recovery following surgery is only 50%, and its clinical application is limited due to the lack of donors and complications at the donor site (Lin and Marra, 2012). Electrical stimulation (ES), among the most popular non-surgical treatment methods, has been widely studied in the field of tissue engineering, both in preclinical and clinical settings. Numerous studies indicate that low-frequency ES delivered post-operatively has a certain positive effect on the repair of peripheral nerve damage, including nerve crush (Foecking et al., 2012), nerve transection (Geremia et al., 2007), and long-gap nerve defects (Huang et al., 2010) in various types of rodents. For example, ES was found to have the ability to accelerate nerve regeneration in combination with steroids, such as testosterone propionate (Sharma et al., 2010). For another example, combined with an reduced graphene oxide (rGO)-coated poly (l-lactic acid-*co*-caprolactone) (PLCL) microfiber scaffold, ES enhanced neurite outgrowth and alignment of PC-12 cells and primary mouse hippocampal neurons compared to control without ES stimulation (Wang et al., 2020). However, it is important to note that the directionality of the electric field exhibited little contribution to neurite alignment, especially for the neurites outgrowth on PLCL fibers with higher diameters (Wang et al., 2020), instead, it has been observed to enhance nerve fiber growth in random and lead axonal misdirection to incorrect end organs which consequently impaired functional recovery (Gordon and English, 2016). Therefore, other interventions such as tissue-engineered tubular structures, i.e., nerve guidance conduits (NGCs) were employed, which have been widely researched in terms of structural design, materials, and fabrication processes, aiming to provide multi-cuefor neural regeneration (Vijayavenkataraman, 2020; Park et al., 2022). Biocompatible and biodegradable materials with appropriate mechanical properties and desirable conductivity are highly beneficial for the establishment of NGCs in peripheral nerve regeneration. By the application of ES, conductive polymer scaffolds have a good effect on differentiation of nerve stem cells and remyelination of regenerated axons (Ghasemi-Mobarakeh et al., 2009; Song et al., 2019; Sun et al., 2019). However, the artificial polymers with excellent electrical conductivity are non-biodegradable, non-soluble, or brittleness, which inhibits them from clinical translation.

Graphene (Gr) and its derivatives graphene oxide (GO) and rGO possess numerous extraordinary properties for use in tissue engineering of the nervous system (Shin et al., 2016; Bai et al., 2018; Raslan et al., 2020; Bellier et al., 2022). As building blocks, they can assemble into various forms of graphene-based scaffolds (GBSs), such as coating, films/membranes, fibers, foams, hydrogen, conduit, 3D printing and bioprinting products. Combined with ES, the GBSs exhibit a particularly brilliant performance for the treatment of PNI (Chen et al., 2019; Dong et al., 2020; Lu et al., 2023). However, ES through conductive GBSs remains in its infancy based upon the fact that effective and safety of parameters of ES need to be confirmed; related mechanism by which ES and GBSs enhance nerve regeneration and the limitation in PNI repair need to be clarified and overcame. Therefore, this short review starts with a brief understanding the characteristics of PNI and the role of ES in repairing injured nerves, followed by a discussion on recent research advances in the preclinical phase of combining GBSs with ES in promoting nerve regeneration. The GBSs are categorized in view of the assemble precursors, i.e., Gr [including chemical vapor deposition (CVD)-G], GO, and rGO nanosheets, which are given in Table 1. This review attempts to elucidate the features of ES imposed by GBSs and prospect the application of GBSs combined with ES in the area of PNI.

**TABLE 1:** Graphene-based scaffolds (GBSs) combined with electrical stimulation (ES) for repairing injured peripheral nerve.

GBMs	Biomaterial(s)	Construct	Electrical conductance	Cell(s)	Animal model	ES parameters (frequency/intensity/duration/number)	Effects	Related mechanism	Ref.
Graphene (Gr)	LIG, PPy	Film	∼0.1–0.5 S cm^−1^	PC-12	NI	50 Hz/400 mV·cm^−1^/2 h-4 h-8 h d^−1^/once	The growth, proliferation and differentiation of PC-12 cells on LIG/PPy electrodes was significantly enhanced by applying ES in view of neurite outgrowth length and neural phenotype	ES induced more protein binding and strengthening cell adhesion and growth	Liu et al. (2022)
	Graphene, AP, PCL, PCLF, ESM	Three-layered conduit with well-defined anisotropy	37.64 ± 0.4 Ω @ 20 Hz	PC-12	NI	0.5 V, 0.03 mA/60 min/once	ES had a positive effect on outgrowth, metabolic activity, the arrangement and morphological changes of PC-12 cells cultured on the tubular scaffolds	NM	Golafshan et al. (2018a)
	Graphene, SA, PVA	Aligned fibrous scaffold	NI	PC-12	NI	1 V/60 min/once	ES facilitated to promote the metabolic activity and proliferation of PC-12 cells	NM	Golafshan et al. (2018b)
	Graphene, PMMA	Film	NI	PC-12	NI	Cell viability: 2 V/1 min, 0.1 s (on-time), 0.01–5 s (off-time); Released dopamine: 1 Hz–10 kHz/2 V/10 s (on-time/off-time = 1)/once	Developed a graphene neurointerface device as a new platform for simultaneous neurotransmitter sensing and neurostimulation for therapy; confirmed the feasibility of graphene for electroceutical applications to various central nerve system disorders	ES increased the intracellular calcium level and facilitated the extracellular dopamine release	Jung et al. (2019)
	Graphene, PANI	Membrane	NI	PC-12	NI	±500 mV/3 h d^−1^/3, 5, and 7 times	PANI-Gr electrode possessed high electro-activity, excellent mechanical and electrical properties, and a high biocompatibility; ES enhanced the axon length of PC-12 and wound regeneration, with no adverse impact on cell density	NM	Zheng et al. (2019)
	Graphene, TPU	Membrane	33.45 ± 0.78 S m^−1^	RSC-96	NI	10, 50, and 100 mV/1 h d^−1^/5 times	The conductive composite membrane was favorable for the viability, growth, and proliferation of SCs stimulated under 10 mV DC voltage	NM	ti
	Graphene, Collagen, PCL (GCFS)	Conduit	3.12 ± 0.62 S m^−1^	MSCs	Rat sciatic nerve (10 mm)	*In vitro*: 2 Hz/10, 20, and 50 mV·cm^−1^/10 min d^−1^/3, 7 times	Combined with ES, GCFS conduit promoted sciatic nerve regeneration and functional recovery	ES facilitated sciatic nerve regeneration by recruitment of endogenous MSCs and modulation of macrophage phenotypes	Dong et al. (2020)
						*In vivo*: 2 Hz/200 mV mm^−1^/10 min d^−1^/14 times			
	Graphene, PCLF, CNTs, MTAC	Hollow conduit	∼10^6^–10^5^ Ω @ 10^3^–10^6^ Hz	PC-12	NI	20 Hz/100 mV mm^−1^/2 h d^−1^/7 times	ES increased number of neurite protrusions in PC-12 cells	NM	Sun et al. (2021)
	Graphene, PLCL, PDA	Micropatterned film	0.0035 ± 0.0004 S m^−1^	RSC-96	Rat sciatic nerve	*In vitro*: 20 Hz/10 mV/1 h d^−1^/3 times	Conduits with ES supported SCs migration, adhesion, and elongation *in vitro*; promoted growth of myelin sheath, faster nerve regeneration, and functional recovery *in vivo*	The groove surface combined with ES enhanced cell adhe-sion and neuronal-specific protein expression	Lu et al. (2023)
		Hollow conduit				*In vivo*: 100 Hz (pulse width = 200 μm, on time = 5 s, off time = 10 s)/30 min d^−1^/14 times			
	Graphene, PGSA, PVP, AgNPs	Flat/Microgroove structure film	∼10^–5^ S cm^−1^ (pure PGSA)	PC-12	NI	50 mV·mm^−1^/2 h d^−1^/7 times	Low cytotoxicity for composites extracts and film with the incorporation of graphene; more and longer neurites outgrowth of PC-12 cells on PGSA-Gr; growth direction of both PC-12 and SW10 cells could be guided by ES; ES enhanced healing rate of cells, the higher electrical conductivity of the PGSA composite films, the higher wound healing rate; faster degradation of the PGSA composite scaffold was observed due to the addition of graphene	NM	Huang and Wang (2023)
			∼10^–4^ S cm^−1^ (PGSA-PVP, PGSA-Gr)	SCs (SW10)					
GO	GO, PPy, DBS, PLLA	Film	32 S cm^−1^	PC-12	NI	50 mV cm^−1^/1 h d^−1^/2 times	ES significantly promotes axonal elongation and arrangement of PC-12 cells	ES enhanced the activity of filamentous filopodia and provided energy to accelerate the actin assembly of growth cone	Shang et al. (2019)
	Carboxylic-GO (C-GO), PPy, PLLA	Film	4.6 S cm^−1^	PC-12	Rat sciatic nerve (10 mm)	20 Hz/1 V/1 h d^−1^//7 times	The incorporation of C-GO improved hydrophilicity of the PPy/PLLA film, and consequently a higher cytocompatibility; functional recovery of ES and conduit group was closer to the autograft group, superior to that of conduit group without ES	NM	Chen et al. (2019)
		Conduit		L929					
	GO, PPy, PDA, PLLA	Film	17.3 S cm^−1^	RSC-96	NI	50 mV cm^−1^/1 h d^−1^/once	Good adhesion to the neural proteins of RSCs; ES arranges 31% of RSCs on the membrane along the current direction	The movement of the cytomembrane proteins under ES and their linkage with serum proteins immobilized by PDA facilitated the extension of growth cone along the ES direction	Li et al. (2020)
	GO, PCL	Fibrous membrane	NI	PC-12	NI	3 Hz/0.5 V cm^−1^/20 min d^−1^/1, 2, 3, and 6 times	Established triboelectric nanogenerators with excellent output performance based on modification of GO nanosheets; *in vitro* ES experiments demonstrated considerable proliferation and migration of PC-12 cells from receiving an alternating electrical field	NM	Parandeh et al. (2020)
	Annealed GO (a-GO), COL	Coating with crumpled surface morphology	1 × 10^6^ Ω/sq	PC-12	NI	23.6 Hz/30–80 mV·mm^−1^/1 h d^−1^/5 times	The coating improved neuronal cell differentiation; facilitated the development of a biohybrid retinal implant that integrated neuronal cells; printed aGO-COL micropatterns supported the creation of neuronal cell microarrays with specific patterns	NM	Yang et al. (2022)
rGO	rGO ink, polyimide	Printed coating	<1 kΩ/sq	MSCs	NI	50 Hz/100 mV/10 min d^−1^/15 times	MSCs differentiated into SC like phenotypes by applying ES from rGO-based electrodes	Electrical stimuli provided by the graphene IDE significantly enhanced the paracrine activity of MSCs and the degree of MSCs’ transdifferentiation	Das et al. (2017)
	rGO, silk fibroin (SF)	Electrospun mat	NI	PC-12	NI	100 mV/2 h d^−1^/2 times	Adhesion and proliferation were improved in PC-12 cells growing on rGO-coated SF mats with cell viability higher than 95%; rGO coating sole without application of ES could induce differentiation of PC-12 cells to neuronal-like phenotypes, while the neurite outgrowth was more pronounced when electric currents were applied	NM	Aznar-Cervantes et al. (2017)
						100 mV/24 h d^−1^/once			
	rGO, *Ap*F, PLCL	Hollow conduit	4.05 × 10^−2^ S m^−1^	RSC-96, PC-12	Rat sciatic nerve (10 mm)	*In vitro*: 100 mV·cm^−1^/1 h d^−1^/5 times	ES promoted the migration, proliferation, and myelin formation of SCs; induced differentiation of PC-12 cells; repair ability of NGC implantation was similar to that of autologous nerve transplantation	The conductive AP/RGO scaffolds under ES were beneficial to SC myelin gene expression and neurotrophin secretion	Wang et al. (2019)
	rGO, PCL	Fibrous membrane	0.105 S m^−1^	RSC-96	NI	10 mV/1 h d^−1^/5 times	ES combined with orientation topography in rGO-coated scaffolds promoted the expression of local NGF, accelerated the migration of SCs, and improved the proliferation of SCs	NM	Huang et al. (2021)
	rGO, CS, OHEC, asiaticoside liposome	Hydrogel	5.27 ± 0.42 ×10^−4^ S cm^−1^	PC-12	NI	250 mV·cm^−1^/8 h d^−1^/once	The hydrogel was non-toxic and suitable for adhesion and proliferation of PC-12 cells *in vitro*; ES made nerve cells highly differentiated and accelerated nerve regeneration; significant inhibitory effect on the growth and collagen secretion of fibroblasts	NM	Zheng et al. (2020)
				RSC-96					
	rGO, PLA, PPy	Nanofiber membrane	1.46 × 10^−1^ S cm^−1^	PC-12	NI	50 Hz/0, 100, 400, and 700 mV·cm^−1^/0.5 h d^−1^/3 times	ES has a significant promoting effect on the proliferation, differentiation, and axonal growth of PC-12 cells under an electric field intensity of 400 mV/cm	When placed in different ES, the protein adsorption is affected by the surface properties and charge of composite nanofibers, which will influence the subsequent adhesion, growth and pro-liferation of nerve cells	Liu et al. (2021)
	rGO, PDA, PVA	Hydrogel	4.3 × 10^−2^ S m^−1^	PC-12	NI	100 Hz/100 mV·cm^−1^/4 h d^−1^/7 times	Successful long-term growth and proliferation of PC-12 cells encapsulated demonstrated the biocompatibility and noncytotoxicity of the hydrogel; highly efficient neuronal differentiation was observed with or without ES	NM	Chen et al. (2021)
	rGO, PLCL	Microfiber	0.95 S cm^−1^	PC-12, primary mouse hippocampal neurons	NI	100–150 mV·cm^−1^/1 h d^−1^/14 times	ES and rGO-coated microfiber with tailored architecture significantly induced orientated neuronal-like network formation	NM	Wang et al. (2020)
	rGO, PCL	Nanofibrils (NF)	0.0443 ± 0.0004 S m^−1^	PC-12	Rat sciatic nerve (5 mm)	100 Hz/100 mV·cm^−1^/1 h d^−1^/7 times	ES stimulated neurogenic differentiation of PC-12 cells; tailored to repair PNI by NGC filled with rGO-coated NF and ADSC	30rGO@NF and ES synergistically facilitated the differentiation of the PC-12 cells into the middle and late stages	Mao et al. (2023)
		Filled conduit							

Abbreviations: AO/EB, acridine orange/ethidium bromide; ADSC, adipose-derived stem cell; AP, alginate-polyvinyl alcohol; ApF, *antheraea pernyi* silk fibroin; AP/RGO, scaffold, coated the rGO, onto an ApF/PLCL, nanofiber; CCFs, conductive composite film; CFGO, carboxyl functionalized graphene oxide; CGO, carboxylic graphene oxide; CNTs, carbon nanotubes; COL, collagen; CS, chitosan; CV, cyclic voltammograms; DBS, sodium dodecyl benzenesulfonate; DC, direct current; ES, electrical stimulation; ESM, eggshell membrane; GBMs, graphene-based materials; GCFS, graphene-based conductive fiber scaffold; GO, graphene oxide; IDE, interdigitated electrode; LIG, laser-induced graphene; LSCM, laser scanning confocal microscope; MSCs, mesenchymal stem cells; MTAC, [2-(methacryloyloxy)ethyl]trimethylammonium chloride; NGC, nerve guidance conduit; NGF, nerve growth factor; NI, not investigated; NM, not mentioned; OHEC, oxidized hydroxyethyl cellulose; PANI, polyaniline; PCL, poly (ε-caprolactone); PCLF, polycaprolactone fumarate; PC-12, rat pheochromocytoma cell line; PDA, polydopamine; PLA, polylactic acid; PLCL, Poly (L-lactic acid-co-caprolactone); PLLA, poly-L-lactic acid; PMMA, Poly (methyl methacrylate); PPy, polypyrrole; PVA, polyvinyl alcohol; rGO, reduced graphene oxide; RSCs, rat Schwann cells; SA, sodium alginate; SCs, Schwann cells; TPU, thermoplastic polyurethane; 3D, three-dimensional.

## 2 Characteristics of peripheral nerve injury

In the peripheral nervous system, a peripheral nerve is wrapped by three layers with different constituents and functions, namely, the endoneurium, perineurium, and epineurium (Liu et al., 2018). According to the Sunderland grading system (Sunderland, 1951), PNI is categorized into five types which sorts the nerve injury into five different degrees, and provides a reference for whether surgical intervention is needed: (I) temporary or reversible block (no surgical intervention required/-); (II) axons are damaged, but the endoneurium, perineurium and epineurium entire (−); (III) axons and the endoneurium are damaged, but the perineurium and epineurium are complete (−); (IV) axons, endoneurium, and the perineurium are damaged, but the epineurium is intact (surgical intervention is necessary); (V) severe nerve injury, with nerves divided into two parts (surgical intervention and nerve transplantation are necessary). The selection of repair methods after injury is usually based on the type of nerve injury, the type of target organ, the selectivity of transplanted nerve donors, the location of nerve injury, and the time interval of nerve injury (Schmidhammer et al., 2022).

**FIGURE 1:**
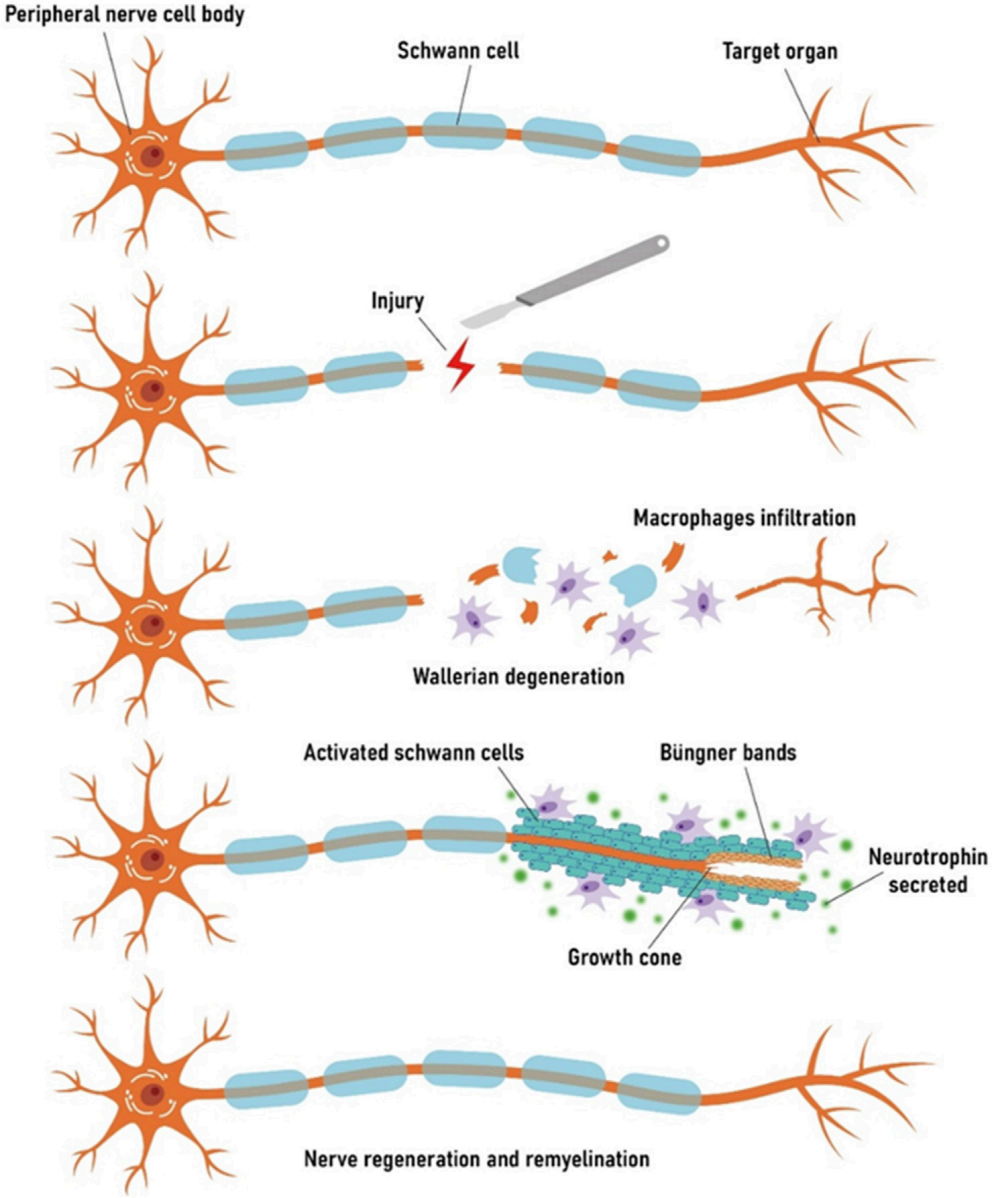
Schematic representation of degeneration and regeneration after PNI.

After PNI, various metabolic, genomic, and biological mechanisms involved in the regeneration of damaged nerve’s structure and function take place (Stoll and Müller, 1999). The destruction of the integrity of the axonal plasma membrane causes a large influx of extracellular calcium and sodium ions into the cytoplasm, leading to the generation of high-frequency action potentials in the proximal axonal region of the cell body, which can retrograde to the cell body (Knott et al., 2014; Rishal and Fainzilber, 2014). Under the mediation of calcium ions (Ca^2+^), upregulation of regeneration-associated genes (RAGs) occurs through the cyclic adenosine monophosphate (cAMP) signaling pathway, which is crucial for the formation of growth cones (Bradke et al., 2012; Donnelly et al., 2013; Mar et al., 2014). Simultaneously, Wallerian degeneration, a series of molecular and cellular changes, providing a microenvironment conducive to axonal regeneration and reinnervation is crucial for nerve repair after PNI (Scheib and Hoeke, 2013; Conforti et al., 2014) (Figure 1). The cell fragments generated by Wallerian degeneration are cleared by Schwann cells (SCs) and macrophages. Besides, activated SCs form Bands of Büngner through the injury gap, guiding the proximal growth cone to reach the neural tube, thereby achieving regenerative innervation of the target organ (Gordon and English, 2016; Gomez-Sanchez et al., 2017). The inflammatory response is likewise an important aspect of peripheral nerve regeneration (Klimovich et al., 2021). In the early stage of injury, M1 macrophages (pro-inflammatory) are mainly recruited, which can enhance the inflammatory response and promote tissue necrosis; in the later stage, M2 macrophages (anti-inflammatory) play a crucial role in effectively responding to hypoxia, increasing the expression of vascular endothelial growth factor A (VEGF-A), and leading to the proliferation and migration of endothelial cells to the injured site (Cattin et al., 2015; Li et al., 2023).

The rate of axonal regeneration is quite slow. Furthermore, because of Wallerian degeneration, the nerve fiber tube lacks an internal structure for a long time, leading to collapse of the nerve tube and an increase in collagen fibers inside it, which results in a smaller diameter and increased difficulty of nerve regeneration (Krarup et al., 2017). The staggered growth pattern of nerve regeneration, where the regenerated axons germinate from the proximal stump at different times rather than growing all at once (Höke et al., 2002), further delays the regeneration of neurons post-PNI. Prolonged denervation impairs target organ function, leading to target muscle atrophy and persistent sensory disturbance (neuralgia or neurosensitivity), which causes long-term distress for patients (Stassart et al., 2013). Therefore, accelerating axonal regeneration speed and reducing the mismatch of regenerated nerve fibers represent the focal points of current research.

## 3 Application of ES for treatment of PNI

As soon as the PNI occurs, endogenous electric fields are generated correspondingly, which may take part in regulating the rate of nerve sprouting, growth, and regeneration. Applied electric fields have likewise been found to influence the regeneration of nerves after injury, i.e., promote survival, migration, and axonal elongation of neurons, either applied immediately following nerve repair or a perioperative ES (Zarrintaj et al., 2020; Kowtharapu et al., 2021; Trueman et al., 2022). In 1952, Hoffman was the first to apply ES to injured nerve. In his study, a 50–100 Hz sine-wave ES was utilized to the injured sciatic nerve of rats for 10–60 min. Results showed that germination was accelerated in the nerves of partially denervated gastrocnemius and soleus muscles (Hoffman, 1952). Subsequently, Pocket and Gavin subjected the sciatic nerve of rats to compression injury and applied ES with a frequency of 1 Hz for 15 min to 1 h, resulting in faster recovery of the toe extension reflex in the ES group (Pockett and Gavin, 1985). To date, there have been few reports of translation of intraoperative ES therapy to the clinic (Roh et al., 2022). In post-operative intervention, for instance, neuromuscular ES, transcutaneous nerve ES), and functional ES have demonstrated potential to alter neuromuscular activity through an electric field (Ni et al., 2023).

**FIGURE 2:**
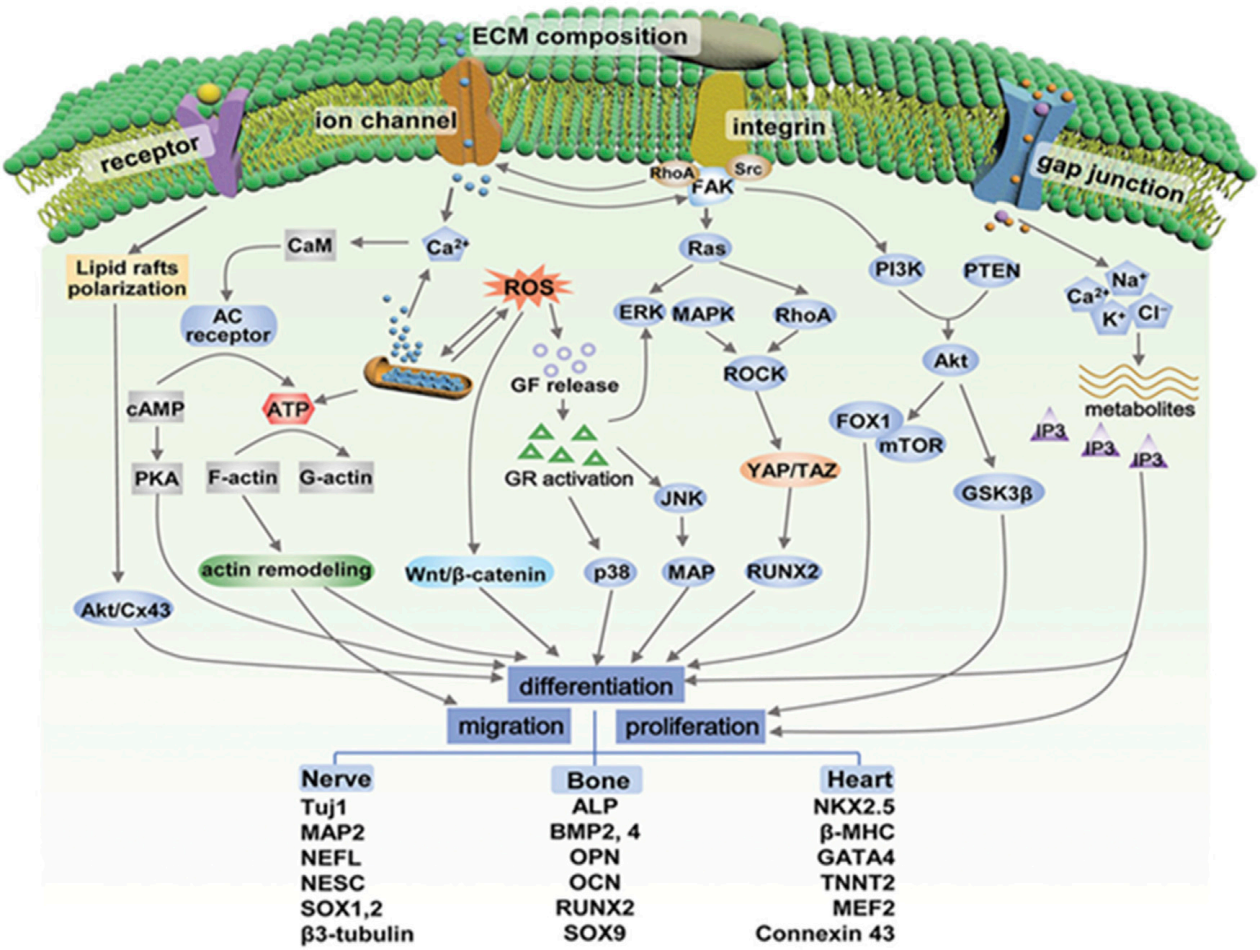
Possible pathways related to biological responses to electrical stimulation. GF, growth factor; GR, growth receptor; CaM, calmodulin; AC, adenylyl cyclase; cAMP, cyclic adenosine monophosphate; PKA, protein kinase A; ERK, extracellular signal-regulated kinase; FAK, focal adhesion kinase; JNK, c-jun N-terminal kinase; MAPK, mitogen-activated protein kinase; PI3K, phosphatidylinositol-3 kinase; PTEN, phosphate and tensin homolog; Src, steroid receptor coactivator; YAP, yes-associated protein; TAZ, transcriptional coactivator with PDZ-binding motif; ROCK, Rho-associated protein kinase; MAP, microtubules-associated protein; mTOR, mammalian target of rapamycin; FOX1: forkhead box protein 1; GSK3β, glycogen synthase kinase-3. Adapted with permission from Ref (Liu et al., 2021). Copyright 2021, John Wiley and Sons.

The mechanism by which ES promotes nerve regeneration is not completely understood. Nevertheless, it is widely believed that it may be related to ES promoting intracellular Ca^2+^ waves, cell membrane potential, membrane receptors, and gap junctions, etc. At axonal injury sites (McGregor and English, 2019; Zuo et al., 2020). The possible pathways related to biological responses to ES are given in Figure 2. For example, *In vivo* studies show that upregulated brain-derived neurotrophic factor (BDNF) due to the increase in Ca^2+^ concentration caused by ES and their high affinity receptor tropomyosin receptor kinase B (TrkB) receptor interactions increase the expression of RAGs, such as T-α-1 tubulin and GAP-43, through the cAMP pathway (English et al., 2007; Wang et al., 2011; McGregor and English, 2019). Subsequently, ES activates cAMP response element binding protein (CREB) through phosphokinase A (PKA), inhibits Rho protein expression in the p75-Nogo receptor (p75-NgR) pathway, and upregulates T-α-1 tubulin, hence enhancing cytoskeleton assembly (Sit and Manser, 2011; Yan et al., 2016). Meanwhile, ES can activate CREB to promote axonal extension through another pathway, namely, the p38 mitogen-activated protein kinase (MAPK) pathway. One study applied ES to PC-12 cells with nerve growth factor (NGF) induced axon growth impairment, and found that the CREB activation pathway could be induced by p38 MAPK to promote axon growth (Kawamura and Kano, 2019). Another study has shown that ES also promoted the induction of pluripotent stem cells into neurons, which may be related to the production of novo ciliary neurotrophic factor (CNTF) (Oh et al., 2021). In addition, *in vitro* experiments indicated that the application of ES (1 Hz, 5 V cm^−1^) promoted the secretion of neurotrophins by SCs, including NGF and NT-3, via the Ca^2+^ influx. Moreover, it has been observed that ES supported the transition of macrophages from M1 to M2, effectively clearing myelin debris, alleviating local inflammatory reactions, and providing a favorable microenvironment for axonal regeneration (Mc Lean and Verge, 2016).

In recent years, the application of electroactive materials in the field of peripheral nerve repair has received increasing attention. These materials not only are capable of connecting damaged nerves in the form of scaffolds, providing mechanical support and physical cues, but also simulate the electrophysiological microenvironment of damaged peripheral nerves and transmit biochemical signals through their own electroactive properties (Wang et al., 2022). However, there still is a long way ahead to repair long-gap PNI under ES conditions. Some possible reasons include 1) The stimulation mode (direct current, alternating current, or capacitive coupling), appropriate timing (pre-, peri-, or post-operative), and parameters of ES protocols including frequency, intensity, time, and number of ES have not been standardized (Gryshkov et al., 2021; da Silva et al., 2020), 2) The exact mechanism by which ES and electroactive materials enhance nerve regeneration is relatively unknown, 3) The misdirection of regenerated axons by ES demands of other interventions such as conduits (Gordon and English, 2016), and 4) the complex circumstances with spatial-temporal evolution of nerve injury *in vivo* become an evident challenge for nervous tissue engineering and regenerative medicine.

## 4 GBSs combined with ES for PNI repair

Since Andre Geim and Konstantin Novoselov first isolated single-layer graphene in 2004, graphene has been a popular material for modern chemistry and physics applications (Novoselov et al., 2004). In recent years, graphene and its derivatives have received extensive attention as a biomaterial for use in the field of tissue engineering and regenerative medicine owing to their variety of extraordinary properties: 1) Controllable mechanical and electrical properties, either as an enhanced coating/blending in a composite or alone, providing mechanical support and physical guidance of extracellular matrix (ECM); 2) Oxygen-containing functional groups or chemical functionalization, endowing them with excellent chemical properties and hydrophilicity, which provide more chemical cues to interacting with cells of PNS; 3) High specific surface area and unique surface features, providing favorable topography and more bioactive sites for cell anchorage and cytoskeletal remodeling; 4) Antibacterial activity, preventing bacterial growth and formation of biofilm on the surface of an implant; 5) Defects and oxidation-dependence of biodegradation; 6) Facilitated fabrication of two-dimensional coatings or three/four-dimensional architectures.

The biocompatibility of graphene and its derivatives has been widely studied, and there are several related reviews on the topic (Kiew et al., 2016; Liao et al., 2018; Amani et al., 2019; Bullock and Bussy, 2019; Kumar and Parekh, 2020). The toxicity of suspended graphene-based materials was found to be highly dependent on their concentration, size of nanosheets, time of exposure, and surface chemistry. Meanwhile, either as a supporting substrate or an implantable medical device, the parameters of the material surface that influence the cellular response are significantly different from their counterparts in suspension (Kumar and Chatterjee, 2016). Reports on the application of GBSs for tissue regeneration as a promising approach found negligible toxicity on cells *in vitro* and in rats for periods as long as 18 months (Kumar and Chatterjee, 2016; Qian et al., 2021). Besides the biocompatibility of graphene and its derivatives for biomedical applications, their biodegradability has also been investigated. In recent *in vitro* and *in vivo* experiments, the biodegradation of GO and rGO was demonstrated to be defect- and oxidation-dependent, which may pave the way for their applications in nanomedicine and biomedical fields (Bellier et al., 2022). However, GO film is unstable in biological solutions and may lead to uncontrollable biosafety issues. Therefore, the incorporation of GO into a matrix-forming composite would be one of necessary choices. Compared with pristine graphene and GO, rGO-based scaffolds are more favorable for electrical active tissue regeneration owing to their high stability in aqueous solutions and remarkable electrical conductivity (Bullock and Bussy, 2019).

The conductive nature of graphene and its derivatives has generated significant interest in neural tissue engineering in recent years (Kumar and Parekh, 2020). They were found to improve proliferation rate of neural stem cells and induce neuronal differentiation, even without the addition of growth factors. In particular, besides electrical conductivity, GO and rGO have the ECM characteristics of PNS, which enables maintenance of high cellular viability, simulates the neurite outgrowth, regulates the degree of neurite extension and number of neuronal branches, and induces axonal alignment with or without adsorbed proteins such as poly (D-lysine) or laminin. Even though there is a great enthusiasm in exploring GBSs in this field and several review papers have been published on the study of GBSs for PNI repair, (Bei et al., 2019; Bellet et al., 2021; Grijalvo and Díaz, 2021; Aleemardani et al., 2022; Chen et al., 2022; Ławkowska et al., 2022), the discussion of ES and graphene-based materials remains only a footnote in the mentioned reviews above. Therefore, considering the significance of ES for neural scaffolds in tissue engineering and regenerative medicine, our review mainly focuses on the current exploration of GBSs combined with ES in PNI, summarizing their respective characteristics and impact on peripheral nerve repair, aiming to give guidance to current clinical potential.

### 4.1 Graphene

**FIGURE 3:**
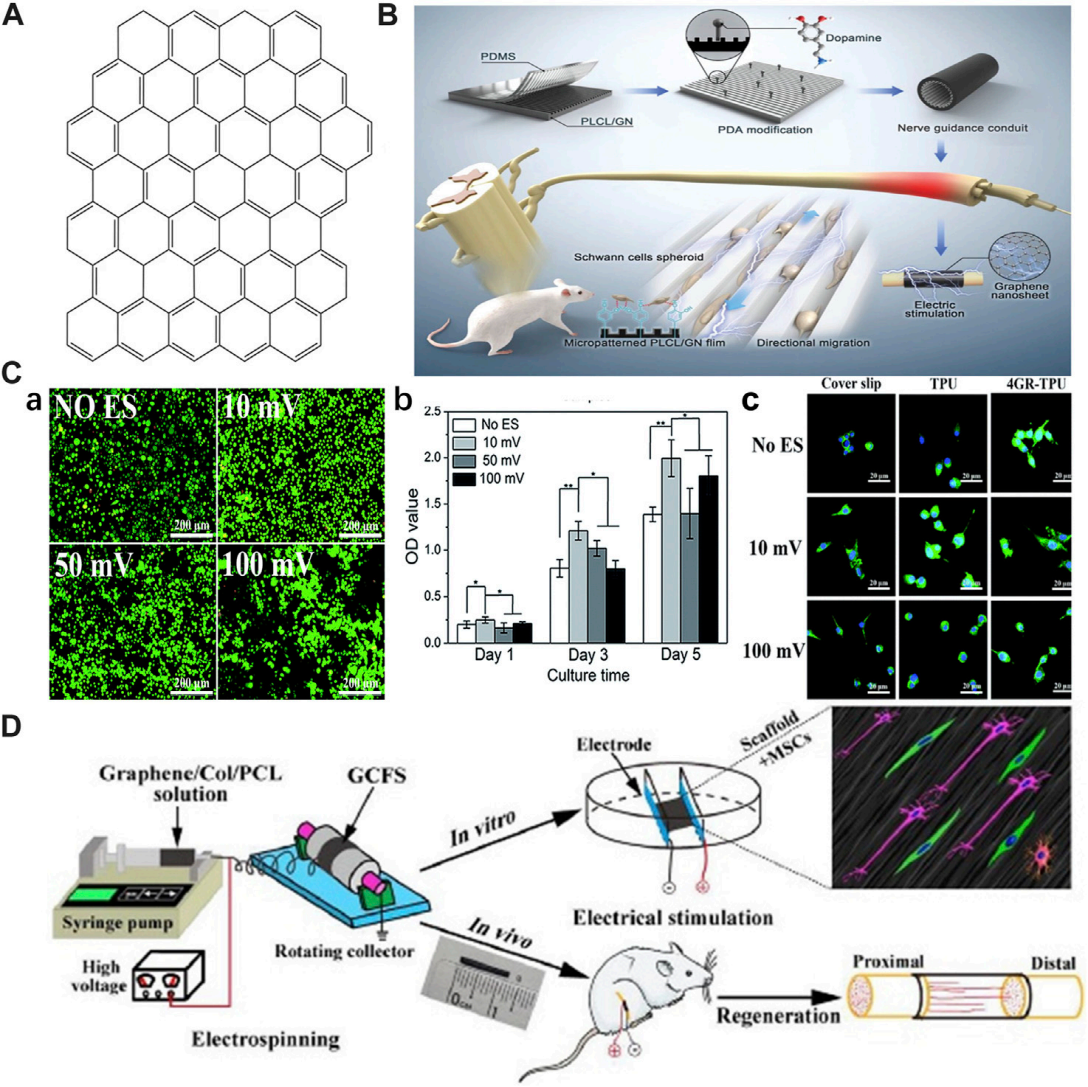
**(A)** Schematic diagram of graphene. Adapted with permission from Ref (Bellier et al., 2022). Copyright 2022, Springer Link. **(B)** Manufacture of PLCL and GN films with stripe micropatterns and PDA modification and their *in vitro* and *in vivo* applicability to accelerate nerve regeneration. Adapted with permission from Ref (Lu et al., 2023). Copyright 2023, John Wiley and Sons. **(C)** SCs are subjected to ES on graphene/TPU composites, and a direct current of 10 mV is more suitable for the growth and proliferation of SCs. **(a)** SCs stained with AO/EB. **(b)** MTT result for SCs proliferation with different voltage. **(c)** LSCM for SCs with the ES of 10 and 100 mV DC. Adapted with permission from Ref (Huang et al., 2019). Copyright 2019, Royal Society of Chemistry. **(D)** ES accelerates the migration of rat MSCs inoculated with GCFS, and significantly enhances the regeneration and functional recovery of the sciatic nerve implanted with GCFS nerve-guided conduit. Adapted with permission from Ref (Dong et al., 2020). Copyright 2020, Elsevier.

Graphene is usually prepared by a mechanical and chemical exfoliation technique, and graphene films/foams with a single, few-, or multi-layer structure are prepared by CVD method (Kostarelos and Novoselov, 2014). Besides, a 3D printing technique has also been studied to fabricate 3D graphene for neuronal networks or conduit (Qian et al., 2018). Because of its two-dimensional atomic structure (Figure 3A) and unique electron distribution character, graphene has excellent physical and chemical properties, including a large specific surface area (∼2,630 m^2^ g^−1^), higher-than-diamond hardness and elastic modulus almost reaching 1 TPa, good optical behaviors (∼97.4% transmittance), high thermal conductivity (∼5,000 W m^−1^ K^−1^, higher than copper), and high electron mobility (∼2 × 10^5^ cm^2^ V^−1^ s^−1^), even higher than that of carbon nanotubes and monocrystalline silicon (Yin et al., 2015; Zhang F. et al., 2022). The unique electrical properties and excellent electrochemical stability make graphene a good candidate for neuronal applications, including neural regeneration (Li et al., 2011; Bendali et al., 2013). In particular, graphene-based composite materials combined with ES accelerate the growth rate of neurons via induction of Ca^2+^ influx, exhibiting high levels of Tuj1 and MAP2 expressions, which led to the investigation of their potential for neural tissue engineering applications (Feng et al., 2015).

The combination of graphene and ES has shown significant advantages in the proliferation and differentiation of PC-12 cells, where the graphene was either used as a coating (Jung et al., 2019; Liu et al., 2022) or mixture (Golafshan et al., 2018a; Golafshan et al., 2018b; Zheng et al., 2019; Sun et al., 2021; Huang and Wang, 2023) in the polymer matrix to improve mechanical, electrical or biological properties. Along with an oriented topography, the graphene-based scaffold could further improve cell proliferation and growth direction *in vitro* (Golafshan et al., 2018a; Golafshan et al., 2018b; Huang and Wang, 2023; Lu et al., 2023). The *in vivo* implantation of PLCL/PDA/GN conduit into rat sciatic nerve defects, which exhibits both electrical conduction and an axon-guiding surface structure, promoted neural regeneration, myelination, and recovery of motor and sensory functions under the synergistic stimulation of ES (Figure 3B). Besides neurons, applying ES (10 mV, 1 h d^−1^, 3 times) through graphene-based conductive polymers yields a positive influence on morphologies and proliferation of SCs, which plays an important role in the process of peripheral nerve repair. Li et al. prepared a conductive composite membrane composed of graphene and TPU (Huang et al., 2019). The presence of graphene significantly improved the mechanical properties and conductivity of the membrane. By applying various voltages (10, 50, and 100 mV) of direct current (DC) ES (1 h d^−1^, 5 times) to the conductive composite containing SCs, a DC voltage of 10 mV was found to be most suitable for survival, synaptic stretching, and the proliferation of SCs (Figure 3C).

The combination of graphene and ES is likewise of great significance for stem cells orienting into specific cell linages. Dong et al. (2020) reported that graphene-based conductive fiber scaffolds (GCFS) prepared by combining different concentrations of graphene with COL and PCL exhibited concentration-dependent conductivity. After studying the effect of GCFS with different graphene contents on neural differentiation of MSCs, they found that a concentration of 1.0 wt% was more conducive to MSCs differentiation into mature neurons; however, higher graphene contents exhibited potential toxicity to the cells. ES promoted the secretion of MSCs and neurotrophic factors. With external stimulation (2 Hz, 20 mV cm^−1^) of MSCs cultured on the 1.0 wt% GCFS surfaces, a significant promotion of the migration and differentiation into neurons was achieved, even though the promoting effect could not be enhanced with increasing ES intensity. For the *in vivo* study, they applied a nerve guidance conduit (NGC) made of 1.0 wt% GCFS, combined with ES (2 Hz, 200 mV mm^−1^, 10 min d^−1^, 14 times), as a bridging material to the sciatic nerve defect site in rats, achieving satisfactory recovery results. ES was found to promote the regeneration and functional recovery of the sciatic nerve after nerve-guided tube implantation. Current *in vitro* and *in vivo* research on ES combined with GBSs with different electrical conductivity provides a reference for their neural tissue engineering applications (Figure 3D) (Dong et al., 2020).

### 4.2 GO

**FIGURE 4:**
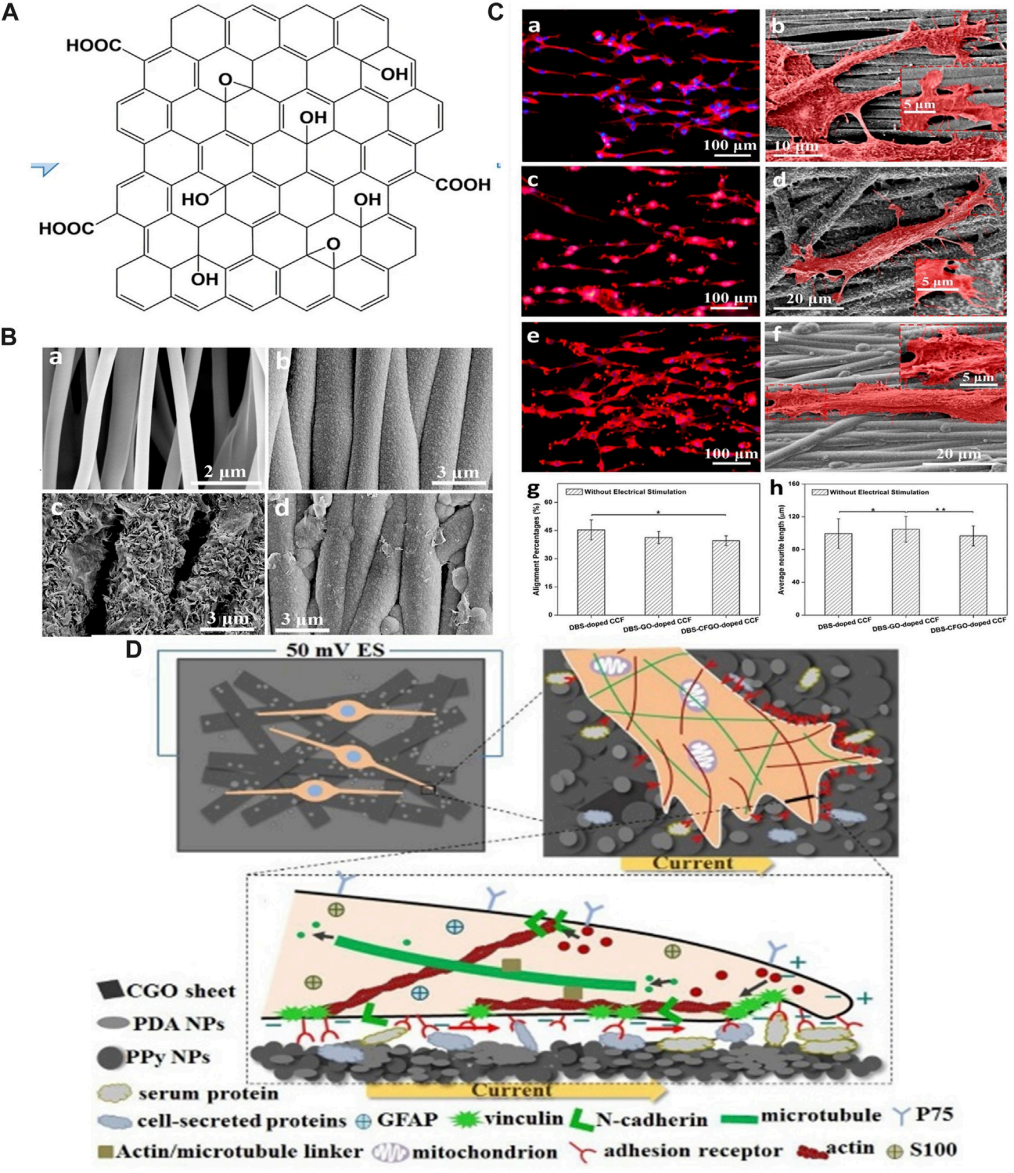
**(A)** Schematic diagram of GO. Adapted with permission from Ref (Bellier et al., 2022). Copyright 2022, Springer Link. **(B)** SEM images of **(a)** PLLA fiber-film, **(b)** DBS-doped CCF, **(c)** DBS-GO-doped CCF, **(d)** DBS-CFGO-doped CCF. Adapted with permission from Ref (Shang et al., 2019). Copyright 2019, American Chemical Society. **(C)** Immunofluorescent images and SEM images of neurites from PC-12 cells on three CCFs with ES: **(a,b)** DBS-doped CCF, **(c,d)** DBS-GO-doped CCF, **(e,f)** DBS-CFGO-doped CCF. **(g)** Neurite alignment percentage. **(h)** Neurite length of PC-12 cells. Adapted with permission from Ref (Shang et al., 2019). Copyright 2019, American Chemical Society. **(D)** ES promotes the alignment of SCs along the current direction on PDA/CGO/PPy PLLA membranes. Adapted with permission from Ref (Li et al., 2020). Copyright 2020, Elsevier.

GO is an oxidized graphene derivative produced by oxidizing graphite with sulfuric acid and potassium permanganate under acidic conditions (Dikin et al., 2007). The surface of GO sheets contains oxygen-containing functional groups such as epoxy, carboxyl, and hydroxyl groups (Figure 4A), which give it good hydrophilicity and colloidal stability, making them more suitable for adhesion, proliferation, and differentiation of cells. Simultaneously, oxygen-containing groups enable it to interact with biological molecules such as peptides, DNA, or proteins through physical adsorption or chemical binding that can be modified or functionalized (Sanchez et al., 2012; Shin et al., 2016). As one of the derivatives of graphene, GO also has unique physicochemical properties for biomedical applications, including as scaffolds in regenerative medicine (Raslan et al., 2020). Even though GO is considered to be an electrically insulating material, its electrical conductivity and piezoelectric property can be regulated by its density and types of oxygen-containing groups, and combining with ES, GO has also been explored for use in peripheral nerve repair (De et al., 2022).

Huang et al. prepared polypyrrole (PPy) conductive composite films (CCFs) doped with GO nanosheets on aligned poly-l-lactic acid (PLLA) fibers using the electrochemical deposition method (Shang et al., 2019; Li et al., 2020). PC-12 cells cultured on the surface of CCFs with administered ES at an intensity of 50 mV cm^−1^ showed significantly higher neurite length and percentage of alignment than those without ES (Figures 4B, C). The enhanced promotion of neurite elongation and orientation was ascribed to GO sheets that coated the surface of the film, providing electrical and topographical cues for regulating PC-12 cells behavior. In another study, *in vitro* experiments were carried out on RSC-96 cells (Li et al., 2020). The results indicated that the film had a promoting effect on the expression of neural proteins, and the application of 1 h ES (50 mV cm^−1^) arranged SCs along the current direction, which was of great significance for peripheral nerve repair (Figure 4D). Furthermore, the study confirmed the synergistic stimulation of ES and conductive conduit with high tensile strength and aligned surface morphology on nerve regeneration and functional recovery (Chen et al., 2019).

### 4.3 rGO

**FIGURE 5:**
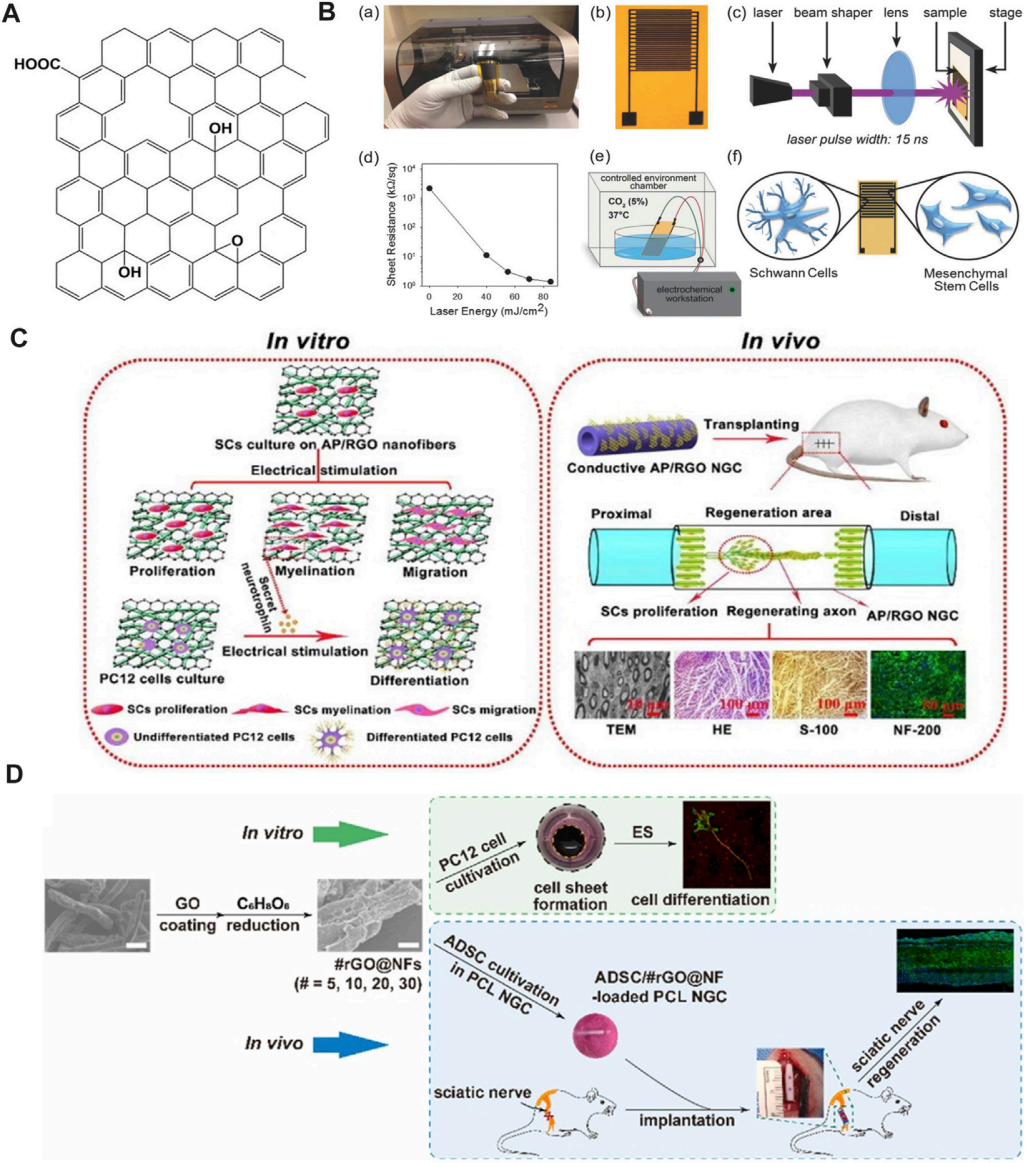
**(A)** Schematic diagram of rGO. Adapted with permission from Ref (Bellier et al., 2022). Copyright 2022, Springer Link. **(B)** Preparation of graphene IDEs and differentiation of MSC under ES. Adapted with permission from Ref (Aznar-Cervantes et al., 2017). Copyright 2017, John Wiley and Sons. **(C)** AP/rGO scaffold enhances the migration, proliferation, and myelin formation of SCs. PC-12 cells cultured on the conductive AP/RGO scaffold exhibit high differentiation after ES. AP/RGO neural guide conduit promotes nerve regeneration *in vivo*. Adapted with permission from Ref (Wang et al., 2019). Copyright 2019, Elsevier. **(D)** PC-12 cells cultivated with rGO-coated NF demonstrate neurogenicity upon ES. Nerve guidance conduit containing the assembly of rGO-coated NF and ADSC promote the recovery of sciatic nerve injury. Adapted with permission from Ref (Mao et al., 2023). Copyright 2023, Elsevier.

rGO can be produced by reducing GO via chemical or thermal treatment. Therefore, rGO exhibits a similar structure to GO, a two-dimensional nanomaterial comprising single-layer sheets of sp^2^ and sp^3^ hybridized carbons, with the exception of the decreased amount of oxygen-containing functional groups (Figure 5A) (Hui et al., 2022). Due to the removal of oxygen-containing groups, rGO exhibits higher thermal stability, higher electrical conductivity, and lower cytotoxicity compared to GO, which are important properties for neural tissue engineering and regenerative medicine (Bellier et al., 2022). In recent years, the application of rGO combined with ES in PNI repair has gained increasing attention.

The combination of rGO and ES promotes the differentiation of MSCs into SCs. Das et al. (2017) electrically stimulated (50 Hz, 100 mV, 10 min d^−1^, 15 times) MSCs using inkjet-printed rGO-based electrodes. The results showed that ES enhances cellular differentiation more than conventional chemical strategies (Das et al., 2017). The circuit made of rGO ink after pulsed-laser processing displayed high conductivity, with a sheet resistance lower than 1 k Ω/sq (Figure 5B). Research has shown that combining rGO-based biomaterials with ES effectively promotes the maturation and differentiation of neurons and regulates the repair function of SCs, which plays an important role in the formation of a peripheral nerve regeneration microenvironment. Wang et al. (2019) coated the rGO onto the surface of an *Ap*F/PLCL nanofiber scaffold through an *in-situ* redox reaction of GO. *In vitro*, the scaffold significantly promoted SC migration, proliferation, and myelin formation, including myelin specific gene expression and secretion of neurotrophic factors. PC-12 cells cultured on the conductive scaffolds also exhibited high differentiation ability with the aid of ES. The *in vivo* performance of implanting AP/rGO NGC into the sciatic nerve defect of rats was similar to that of autologous nerve transplantation (Figure 5C). Liu et al. prepared conductive PLA/rGO/PPy composite nanofibers by the incorporation of rGO into PLA (Dikin et al., 2007). Owing to the presence of conductive PPy and rGO, the conductivity of the composite achieved 1.46 × 10^−1^ S cm^−1^, which is higher than some of the graphene-based composites, as shown in Table 1. To study the effect of ES on the proliferation and differentiation of nerve cells seeded on scaffolds with high electrical conductivity, the authors applied electric field strength of 0, 100, 400, and 700 mV cm^−1^ and 50 Hz for 0.5 h d^−1^ for 3 days. The electric intensity of 400 mV cm^−1^ was found to be most favorable for cell proliferation, differentiation, and neurite growth. Most recently, Mao et al. (2023) developed a conductive NGC for better nerve regeneration compared to their previous study through filling with PCL NF that was coated by rGO layers. *In vitro* study demonstrated the excellent cytocompatibility of rGO@NF with 30 layers of rGO, which exhibited the highest electrical conductivity and promoted PC-12 cells extension and neurite outgrowth in the presence of ES. Further, transplantation of the NGC *in vivo* to bridge the nerve defect in a Sprague Dawley rat model accelerated nerve regeneration to a greater extent compared to bridging the fractured nerve by a hollow NGC (Figure 5D). All the results above indicate that ES combined with rGO is an efficient strategy to develop an artificial implant for long-gap PNI repair.

## 5 Summary and perspectives

As a common clinical disease, the incidence rate of PNI has experienced an upward trend in recent years. ES is considered an effective treatment for PNI, and has been extensively studied in the preclinical stage. GBSs represent promising media and carriers for ES, owing to their excellent electrical conductivity and mechanical properties. The combination of GBSs and ES has shown encouraging effects in promoting stem cell differentiation, inducing neuronal repair, and promoting the proliferation, migration, and maturation of SCs. However, their practical applications have certain limitations that must be overcome. First, research to date is still limited to the preclinical stage, and most reports concern *in vitro* studies. Meanwhile, a large amount of *in vivo* and clinical translation data is needed to support a next-generation scaffold. Furthermore, there are still challenges in the biocompatibility of GBSs, and long-term safety *in vivo* is of particular significance for GBSs in future clinical applications. In addition, low-frequency or direct current electric fields are currently chosen to administer post-operational ES through GBSs. However, given the importance of balance between conductivity and ES for a proper neural regeneration, stimulation settings of the intensity of the applied ES through GBSs—in particular 3D-graphene or 3D-reduced graphene oxide with superior electrical conductivity must be carefully taken into account. In addition, the potential for GBSs piezoelectricity is still to be extensively explored to provide an effective platform for a wireless or non-invasive repair of PNI.

GBSs have been reported to assist in regulating neuronal excitability, which has a significant impact on neuronal repair and axonal regeneration. Simultaneously, studies indicate that GBSs can serve as a bridge to connect nerve defect sites and help transmit chemical signals between cells, promoting nerve regeneration. Further, reports showed their ability to improve the microenvironment of nerve repair, promote angiogenesis, and regulate immune responses. The GBSs with their unique topography and surface structure exhibit strong effect on the morphology and differentiation of stem cells into neurons. Even though the biodegradation of graphene and its derivatives is still a challenging issue for applications in tissue engineered grafts, the development of neural prosthesis or non-degradable flexible electronics for long-term applications for next stage of nerve guidance conduit or neural electrodes may have opened up numerous opportunities to GBSs for an optimal recovery in patients with PNI.
